# Supplementary material for: Estimating and interpreting secondary attack risk: Binomial considered biased
Source: PLoS Comput Biol. 2021 Jan 20;17(1):e1008601. doi: 10.1371/journal.pcbi.1008601 (PMC7850487; doi:10.1371/journal.pcbi.1008601)
Supplement: Attachment — Submitted filename: comments.pdf [file pcbi.1008601.s006.pdf]

In the manuscript titled “Estimating and interpreting secondary attack risk: Binomial considered harmful”, the authors demonstrate that using widely adopted binomial models when estimating household SAR could lead to biased estimate due to the unrealistic assumption that the primary case is the sole source of infection for all secondary cases within the household, ignoring the likelihood of multiple-generation transmissions. The authors also demonstrate that methods, including longitudinal chain binomial model and pairwise survival analysis, that take into account multi-generation transmissions provide less biased estimates and shall be given preference over binomial model. The authors rightfully point out this unrealistic but commonly tolerated assumption in household studies of infectious disease transmission. The manuscript is well written and supported by well-designed computational experiments. I recommend the manuscript for publication once the following questions and comments are clarified and addressed:

- I recommend the authors avoid using unnecessarily negative word like “harmful” in the title (could be replaced by more neutral word like “biased” or “inaccurate”).
- In Table3, the authors should consider reporting AIC scores for “binomial” and “longitudinal chain binomial” models as well to directly demonstrate “longitudinal chain binomial” and “pairwise regression” models fit the data better than “binomial” model.
- P14, Fig5: When simulating the “binomial model” based on SAR estimates of LA household study, do the authors simulate multi-generation transmissions within the household? (based on Section 2.2, it seems like the procedure would generate multi-generation transmission). Although unrealistic, the “binomial model” assumes no multi-generation transmission when inferencing SAR. Thus, the simulations of household transmission need to be consistent with this assumption so that it can be fairly compared with the “pairwise regression” and “longitudinal chain binomial” models.
- The likelihood of violating “no multigeneration transmission” assumption increases sharply with household size, and we would expect increasing bias for larger household. This is clearly demonstrated in Fig 1 based on simulation exercise. If the authors also demonstrate the effect with the observational data in the LA household study, it would make the arguments of the paper even more convincing.
