# Supplementary material for: Estimating and interpreting secondary attack risk: Binomial considered biased
Source: PLoS Comput Biol. 2021 Jan 20;17(1):e1008601. doi: 10.1371/journal.pcbi.1008601 (PMC7850487; doi:10.1371/journal.pcbi.1008601)
Supplement: Attachment — Submitted filename: Response.pdf [file pcbi.1008601.s007.pdf]

Responses to Reviewer Comments: PCOMPBIOL-D-20-01278

November 7, 2020

We are very grateful to both reviewers for their useful comments. Point-by-point responses are given below. We hope we have addressed their concerns, and we would be happy to make additional revisions if needed.

Reviewer 1

> In the manuscript titled "Estimating and interpreting secondary attack risk: Binomial considered harmful", the authors demonstrate that using widely adopted binomial models when estimating household SAR could lead to biased estimate due to the unrealistic assumption that the primary case is the sole source of infection for all secondary cases within the household, ignoring the likelihood of multiple-generation transmissions. The authors also demonstrate that methods, including longitudinal chain binomial model and pairwise survival analysis, that take into account multi-generation transmissions provide less biased estimates and shall be given preference over binomial model. The authors rightfully point out this unrealistic but commonly tolerated assumption in household studies of infectious disease transmission. The manuscript is well written and supported by well-designed computational experiments. I recommend the manuscript for publication once the following questions and comments are clarified and addressed:

Detailed comments

1. I recommend the authors avoid using unnecessarily negative word like "harmful" in the title (could be replaced by more neutral word like "biased" or "inaccurate").

   We have replaced "harmful" with "biased", and we agree that this more accurate and less inflammatory.

2. In Table3, the authors should consider reporting AIC scores for "binomial" and "longitudinal chain binomial" models as well to

> directly demonstrate "longitudinal chain binomial" and "pairwise regression" models fit the data better than "binomial" model.

The AIC is a valid comparison between models that are fit to the same data, as are the different parametric pairwise survival models. Because the binomial GLM models do not use person-time data, the AIC cannot be used to compare the binomial models to the the pairwise survival or chain binomial models. The binomial GEE models use a quasi-likelihood, so the AIC is not generally applicable for model comparison. To emphasize that the AIC is being used only to compare the parametric pairwise survival models, we have added the following sentence to the description of Table 3:

> To compare goodness-of-fit among the parametric pairwise survival models, we used the Akaike Information Criterion (AIC).

The difference in goodness-of-fit between the binomial models and the other models is demonstrated in the histograms in Figures 5–7. These show that the SAR estimates from the binomial model are too high, which is the expected result based on the arguments made in the manuscript.

3. P14, Fig5: When simulating the "binomial model" based on SAR estimates of LA household study, do the authors simulate multi-generation transmissions within the household? (based on Section 2.2, it seems like the procedure would generate multi-generation transmission). Although unrealistic, the "binomial model" assumes no multi- generation transmission when inferencing SAR. Thus, the simulations of household transmission need to be consistent with this assumption so that it can be fairly compared with the "pairwise regression" and "longitudinal chain binomial" models.

The simulation test needs to capture important features of within-household transmission whether or not these conform to the assumptions of the statistical models. The assumption of no multi-generation transmission is generally understood to be an approximation, not an assertion that secondary infections are actually not infectious. Our goal here is to show that this approximation is far worse than expected, so the simplifying assumption made by all binomial models is not justified in practice. To clarify this important point, we have added the following sentence to the first paragraph of Section 2.2:

> This simple model allows but does not require multiple generations of infection within households, allowing us to evaluate the quality of binomial estimates that make the simplifying assumption of a single generation of transmission.

4. The likelihood of violating "no multigeneration transmission" assumption increases sharply with household size, and we would

> expect increasing bias for larger household. This is clearly demonstrated in Fig 1 based on simulation exercise. If the authors also demonstrate the effect with the observational data in the LA household study, it would make the arguments of the paper even more convincing.

This is an interesting suggestion, and we conducted analyses at different household sizes. When we tried this, we did not find a meaningful difference between the estimates from the binomial or pairwise models. This is likely due to the concentration of household sizes (29 out of 58 have sizes 3–5), the small sample size, and the complexity of the relationship between the SAR and FAR under realistic conditions (see response #3 to Reviewer 2 below).

Reviewer 2

> This manuscript highlights the risk of using binomial models to estimate the secondary attack risk in clusters and highlights the importance of accounting for multiple generations of cases. This is a well-know problem for mathematical epidemiologists that, unfortunately, it is much less clear to more traditional epidemiologists. I believe this manuscript deals with a very important issue and provides a clear and accessible way to understand it for traditional epidemiologists. The theoretical framework looks solid to me, while the analysis of the LACDPH household data has a key flaw that should be addressed (see below).

Detailed comments

1. When analyzing the LACDPH household data, the authors are assuming that all influenza infections will result in an acute febrile respiratory illness. There are several studies showing that the probability of developing fever after influenza infection is ¡30% - I would like to point the authors to Carrat et al, Am J Epidemiol, 2008 and references therein. Therefore, it is very likely that the LACDPH missed more than half of the influenza infections. I agree with the authors' choice to keep the transmission model as simple as possible and not include other factors such as age-specific susceptibility to infection, age-specific infectiousness, etc. However, the probability of developing fever is so important to proper interpreting the LACDPH household data that that cannot be neglected in the simulation analysis. As such all claims that some cases cannot be explained unless the re-importation of the infection to the household should be removed (e.g., line 279, 346). Also the definition of "late case" (line 276) should be revisited accordingly.

This is a very important point. We have revised the definition of "late case" to read:

> *Late cases* are susceptible household members who were infected after the end of the infectious period of the last final size case in the household. Given the assumed infectious period, these cases can only be explained by a new introduction of infection to the household or by transmission paths that include undetected cases. In volunteer challenge studies, approximately 71% of influenza A (H1N1) infections result in symptoms and 37% result in fever $\geq 100°$F [55].

We have included the reference to Carrat *et al.* (Ref. 55), and we have corrected all later discussion to include the possibility that late cases are infected through transmission paths that include undetected cases.

2. As for most infectious diseases, the duration of the infectious period of influenza is unknown and we have only rather indirect estimates of it. Therefore, I agree with the authors' idea of exploring a wide range of values. However, I see two issues here. First, the list of explored values is way too large. Second, the distribution is clearly not uniform. We have a wide range of studies showing that the mean generation time of influenza is about 2-4 days. An infectious period of 12 days would be possible only if the transmission probability after the very first few days is extremely low. This should be clearly discussed and I suggest to use a more realistic (shorter) value for the infectious period in the baseline analysis and to decrease also the value of the upper bound as 12 days appear to be highly unrealistic.

We have revised the LA households simulations to use 4-day, 6-day, and 8-day incubation periods, which are more consistent with a mean generation interval of 2–4 days. Based on Yang et al. (2009), our primary analysis retains the six-day infectious period.

3. I think it would be very interesting to look at the FAR by household size in the LACDPH and provide a comparison with the obtained modeling results. In fact, I fear that the model may fail the comparison with the data in this respect. If so, this should be clearly stated and acknowledged as a study limitation possibly linked to the many additional factors that are not included in the simple models used here.

We tried to calculate the binomial estimate of the SAR separately in small and large households (see response #4 to Reviewer 1 above). We did not see a meaningful difference due to the small sample size and concentration of household size distributions. The theoretical model analyzed using PGFs was not meant to be a realistic representation of the LACDPH households; it was a simple demonstration that the approximation used by

binomial estimates of the SAR is less accurate than commonly thought. The FAR of the LACDPH households is not clearly defined because of variation in household sizes, co-primary cases, the ongoing risk of infection from outside the household, and variation in susceptibility and infectiousness within and between households. We have revised and rearranged the Discussion to clarify these points.

4. Line 61-62. As stated before, the duration of the infectious period of influenza is unknown. The same applies also to the latent period. What we do know are the length of the generation time (mean roughly in the range 2-4 days) and of the incubation period (mean roughly in the range 1-2 days). I strongly recommend rephrasing this sentence in terms of incubation period and generation time that would better support the (correct) authors reasoning here.

We have rephrased this in terms of the incubation period and the duration of viral shedding with references to Carrat et al. (AJE, 2008) and Longini et al. (Science, 2005).

5. Line 71-72, "so the binomial [...] of the SAR". I recommend dropping this part of the sentence.

Done.

6. Line 331. I suggest dropping the reproduction number from the list given here.

Done.

7. There are a few very minor English mistakes here and there (e.g., lines 47, 55, 57).

These have been corrected, and we proofread the entire manuscript again to catch as many of these errors as we can.
